# Myocardial Crypts on Ultrasound in a Young Female with Exertional Syncope

**DOI:** 10.5811/cpcem.48812

**Published:** 2026-03-13

**Authors:** Christopher Allen, Alexandra Gubbels, Youyou Duanmu, Jody Vogel

**Affiliations:** Stanford University, Department of Emergency Medicine, Palo Alto, California.

**Keywords:** myocardial crypts, genetic cardiomyopathy, hypertrophic cardiomyopathy, point-of-care ultrasound, syncope

## Abstract

**Case Presentation:**

A 20-year-old female with no past medical history presented to the emergency department (ED) after an episode of exertional syncope. Physical examination, vital signs, and electrocardiogram were unremarkable. Point-of-care ultrasound revealed abnormal invaginations in the interventricular septum. Laboratory evaluation was significant for markedly elevated troponin concerning for cardiac arrest. She was admitted to cardiology with suspicion for genetic cardiomyopathy. The patient underwent placement of an implantable cardioverter defibrillator after cardiac magnetic resonance imaging redemonstrated the septal invaginations known as myocardial crypts. Genetic studies later revealed sarcomere gene mutations associated with hypertrophic cardiomyopathy.

**Discussion:**

Myocardial crypts, which are invaginations within the myocardium, are considered early morphological markers for hypertrophic cardiomyopathy and may precede the development of overt hypertrophy. The presence of myocardial crypts and syncope is highly concerning for evolving hypertrophic cardiomyopathy. In this case, identifying myocardial crypts on ED point-of-care ultrasound, in conjunction with clinical context, facilitated further confirmatory diagnostics and timely intervention with placement of an implantable cardioverter defibrillator.

## CASE PRESENTATION

**Image 1:**
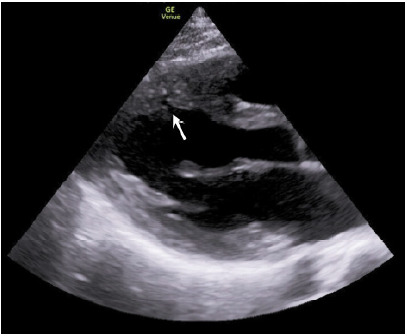
Point-of-care ultrasound echocardiogram on parasternal long-axis view demonstrating an invagination within the anteroseptal wall of the interventricular septum, known as a myocardial crypt (arrow). No septal hypertrophy, left ventricular outflow tract obstruction, or other structural or kinetic abnormalities were appreciated.

A 20-year-old female presented to the emergency department (ED) after an episode of exertional syncope. She denied chest pain, prodromal symptoms, and past medical history, although she had reported a similar episode of exertional syncope years prior and a family history of sudden cardiac death. Physical examination and vital signs were unremarkable. Electrocardiogram (ECG) showed normal sinus rhythm without evidence of ischemic or dysrhythmic morphologies. Prior to laboratory results, point-of-care ultrasound (POCUS) echocardiogram (echo) revealed abnormal invaginations in the interventricular septum on parasternal long-axis view (Image 1).

**Image 2:**
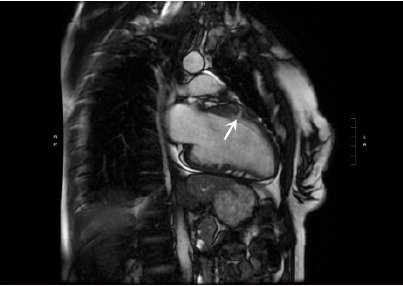
Cardiac magnetic resonance imaging confirming myocardial crypt within the anteroseptal wall (arrow). On final impression, there were two additional myocardial crypts noted within the inferoseptal wall. Otherwise, there was normal left ventricle and right ventricle wall thickness, wall motion, and ejection fraction.

While no obvious ventricular septal hypertrophy, left ventricular outflow tract (obstruction, or other structural or kinetic abnormalities were appreciated on POCUS, the septal invaginations raised suspicion for a structural cardiac cause of this patient’s syncope. Laboratory evaluation demonstrated markedly elevated troponin (1,961 nanograms per liter (ng/L) (reference range 0–10 ng/L for females) with leukocytosis, transaminitis, and acute kidney injury concerning for preceding cardiac arrest as etiology of syncope. These findings, including POCUS echo, were conveyed to the inpatient cardiology team who admitted the patient for telemetry and further diagnostics including cardiac magnetic resonance imaging (MRI). Cardiac MRI redemonstrated previously seen septal invaginations on echo, and formal radiographic interpretation confirmed these to be myocardial crypts (Image 2) without ventricular hypertrophy, wall motion abnormalities, or other abnormal findings.

Ultimately, the patient underwent implantable cardioverter defibrillator placement for presumed diagnosis of genetic cardiomyopathy and was discharged after an uneventful postoperative course. Genetic studies later confirmed this diagnosis, with results demonstrating sarcomere mutations associated with hypertrophic cardiomyopathy.

*CPC-EM Capsule*

What do we already know about this clinical entity?

*Myocardial crypts are invaginations in the myocardium, that may indicate early disease in hypertrophic cardiomyopathy. They are usually detected on magnetic resonance image*.

What is the major impact of the image(s)?

*The ultrasound images demonstrate myocardial crypts can be seen on ultrasound, raising suspicion for an underlying cardiomyopathy prior to genetic confirmation. Ultrasound images show myocardial crypts can be seen on point-of-care ultrasound, raising suspicion for cardiomyopathy before MRI or genetic confirmation*.

How might this improve emergency medicine practice?

*Few reports describe myocardial crypts in emergency medicine. Our case highlights their detection on point-of-care ultrasound and the value of awareness for risk stratification and diagnosis*.

## DISCUSSION

Genetic cardiomyopathy refers to a group of disorders caused by inherited mutations affecting the myocardium. The most common type is hypertrophic cardiomyopathy, characterized by ventricular septal hypertrophy leading to impaired hemodynamics from left ventricular outflow tract obstruction. Myocardial crypts are narrow, blood-filled invaginations within the myocardium that while not pathognomonic for hypertrophic cardiomyopathy, can serve as an early morphological marker for hypertrophic cardiomyopathy and may precede the development of overt hypertrophy.^1^–^3^ Myocardial crypts can be visualized on cardiac MRI in approximately two thirds of individuals carrying hypertrophic cardiomyopathy-related gene mutations prior to development of phenotypic hypertrophic cardiomyopathy, and in over half of these cases myocardial crypts precede detectable ECG abnormalities.^4^,^5^

Myocardial crypts can also be visualized on echocardiography. This case is, to our knowledge, the first description of myocardial crypts found on ED-performed POCUS. While myocardial crypts may occasionally be benign, in the appropriate clinical context, their finding on any imaging modality should raise suspicion for genetic cardiomyopathy as cardiology case reports have demonstrated.^6^–^7^ Although hypertrophic cardiomyopathy-related sudden cardiac death occurs at an estimated 1% per year, early detection in the ED is challenging, with approximately 10% of patients lacking characteristic ECG abnormalities.^8^,^9^ This increases the utility of POCUS; unlike cardiac MRI, POCUS is widely available to the emergency physician when there is concern for hypertrophic cardiomyopathy. For this patient, recognizing myocardial crypts on POCUS, in conjunction with clinical context and laboratory data, raised suspicion for an underlying myocardial disorder leading to further confirmatory diagnostics and timely intervention with placement of an implantable cardiac defibrillator for prevention of sudden cardiac death.

## CONCLUSION

This case highlights the potential role of POCUS in identifying subtle structural cardiac abnormalities in patients presenting with high-risk syncope. The detection of myocardial crypts on ED POCUS, even in the absence of septal hypertrophy or ECG abnormalities, raised suspicion for an underlying genetic cardiomyopathy and prompted expedited diagnostic evaluation. In patients with exertional syncope and concerning historical features such as a family history of SCD, recognition of myocardial crypts may represent an early imaging clue for evolving HCM. Incorporating careful assessment of myocardial morphology during ED POCUS may therefore help identify patients who warrant further cardiology evaluation and risk stratification.
